# Prevalence and associated factors of anemia among full-term newborn babies at University of Gondar comprehensive specialized hospital, Northwest Ethiopia: a cross-sectional study

**DOI:** 10.1186/s13052-019-0764-1

**Published:** 2020-01-03

**Authors:** Tegenaw Tiruneh, Elias Shiferaw, Bamlaku Enawgaw

**Affiliations:** 1 Department of Medical Laboratory Sciences, College of Medicine and Health Sciences, Debre Tabor University, Debre Tabor, Ethiopia; 2 Department of Hematology and Immunohematology, School of Biomedical and Laboratory Sciences, College of Medicine and Health Sciences, University of Gondar, Gondar, Ethiopia

**Keywords:** Full-term newborn, Newborn anemia, Umbilical cord blood, Gondar, Ethiopia

## Abstract

**Background:**

Anemia in newborn babies causes asymptomatic to an acute life-threatening event. If untreated, it leads to a delay in brain maturation, tissue hypoxia, and stunted growth. In Sub-Saharan Africa, its burden ranges 23–66%. However, in Ethiopia, there is limited information regarding the prevalence and associated factors of newborn anemia. Thus, this study was aimed to assess the prevalence and associated factors of anemia among full-term newborn babies.

**Method:**

Cross-sectional study was conducted from February 1 to April 30, 2019, among 192 full-term newborn babies. A systematic random sampling technique was employed to select study participants. Socio-demographic characteristics were collected through interviews. Clinical data were collected by reviewing medical records. Cord blood was collected from the clumped cord. Complete blood count was analyzed by using the Sysmex KX-21 N hematology analyzer. SPSS 20 was used to analyze the data. Bivariable and multivariable binary logistic regression were used to identify associated factors. *P*-value < 0.05 was considered a statistically significant association.

**Result:**

The median (interquartile range) of cord hemoglobin was 15 g/dL (13.93–16.2 g/dL). From the total, 25% (95% CI: 18.9, 31.1%) of the newborns were anemic. From anemic 89.5, 6.3, and 4.2% were mild, moderate and severe anemia type, respectively. Maternal vegetable consumption habit (AOR = 0.34, 95%CI: 0.17, 0.69) were significant associated with anemia.

**Conclusion:**

Anemia among newborn babies found to be a moderate public health problem. Based on the finding early screening of newborn anemia may reduce further complications.

## Background

Anemia is a major public health problem globally. About two billion people suffer from anemia, 68.36 (8.8%) million lived with disability and one million deaths occur in Africa and Southeast Asia. The general prevalence and severity of anemia in low economic countries may reach 50 to 80%, and 10 to 20%, respectively. From this burden, newborn anemia is a serious public health issue by causing infant mortality and morbidity in early neonatal life [1–3]. Its prevalence is very high in sub-Saharan Africa range from 23 to 66% [4, 5].

Anemia in newborn babies has a complex problem and has a unique blood picture due to variation in hematological profiles [6]. It can be severe to the acute life-threatening event [7]. It also causes impairment in brain development, delay in brain maturation, poor school performance and work capacity in later years, stunted growth, tissue and organ hypoxia, poorer cognitive, motor, and social-emotional development [2, 8].

Nutritional deficiency anemia is a common cause of morbidity worldwide in all age groups. It affects an estimated 1 to 2 billion people. From this burden, newborn babies are more vulnerable groups next to pregnant women. It is also more severe in developing countries that have an inadequate iron intake, poor interventional implementation program, and poor infrastructure services for early diagnosis and treatments of anemia at the health facility level [9, 10].

In addition to the above, nutritional deficiency especially iron deficiency is the most common cause of nutritional anemia among pregnant women. Mostly due to deficiency of raw material used for the production of hemoglobin (Hgb). This problem also increased due to increased metabolic demands imposed by pregnancy such as growing placenta, fetus and maternal tissues. Therefore, it increases the risk to develop maternal anemia. Since the fetus depends on the maternal iron level, higher risk women for iron deficiency with concomitant increased risk of anemia among newborn babies. Due to these, anemia in the newborn has serious public health issues in developing countries [11, 12].

The newborn period is the most dynamic period because of profound variations and adjustments during transit of fetus from dependent, hypoxic, intrauterine life to totally independent neonatal life. Even though the fetus is fed and protected by the mother, it may be influenced by maternal nutrition intake and anemia [13]. Therefore, it is mandatory to assess the prevalence and associated factors of anemia among newborn babies at the time of delivery.

## Methods

### Study setting, design and population

An institution-based cross-sectional study was conducted from February 1 to April 30, 2019 at the gynecology and obstetrics department of University of Gondar comprehensive specialized hospital, northwest Ethiopia. University of Gondar comprehensive specialized hospital is located in Amhara region, 180 km far from Bahir Dar, the capital city of Amhara regional state and 727 km from Addis Ababa, the capital city of Ethiopia. The town located 2133 m elevation above sea level. The hospital has a range of specialties including pediatrics, surgery, gynecology, psychiatry, human immunodeficiency virus (HIV) care and an outpatient clinic. A total of 192 full-term newborn babies with their mothers were included in this study. Twin newborns, newborns with inaccessible cord blood sample and complicated pregnancy with diabetics, preeclampsia, hypertension, HIV/AIDS, chronic kidney, liver disease, malaria, any hematological and non-hematological malignancy were excluded from being sampled. Only full-term newborn babies (39–42 weeks of gestation) that fulfilled the above-listed eligiblity criteria were included in the study. A systematic random sampling technique was employed to select study participants.

### Sample size determination

The sample size was determined using single population proportion formula by considering the following assumptions, taking 21% prevalence of anemia from the previous study done in Netherland [14], 95% confidence interval (Z = 1.96), 5% level of significance and 5% maximum allowable error (w = 0.05). Since the study population was less than 10,000, we used the correction formula. Accordingly, the final sample size was 192 newborn babies.

### Sampling technique

The study participants were selected by systematic random sampling technique. Before the selection of study participants, we calculated the average delivery rate of 1 month that is January based on data from maternal registration. Based on this, the average delivery rate was 8.5 per day. Based on the data we estimate the total delivered mothers from February 1 to April 30, 2019, which becomes around 765. This figure was taken as a sampling frame. Then sampling interval (K^th^) was determined by dividing the total estimated delivery from February 1 to April 30 by total sample size and it was 4.

The first study participant was selected randomly by lottery methods between one and K and the next study participant was selected every fourth interval during the flow of delivery until the allocated sample size reached. The excluded study participants were substituted with the next consecutive study participants.

## Data collection method

### Socio-demographic and clinical data collection

A pre-tested structured questionnaire prepared in English and translated to the local language (Amharic) was used to obtain maternal sociodemographic, nutritional status and newborn gender via face-to-face interviews. Maternal clinical data like ANC follow up, supplementation of iron and folic acid during pregnancy, information about the number of pregnancies (gravida) and presence of bleeding during pregnancy were retrieved from maternal medical records with the aid of data extraction sheet.

### Blood collection and laboratory analysis

About 3 ml of UCB specimen was obtained after delivery from the clamped umbilical cord. Two Midwifery professionals collected the cord blood sample from the clamped cord through excluding of the placenta. The collected sample was immediately poured into tri-potassium ethylene diamine tetraacetic acid (K3-EDTA) test tube and gently mixed to prevent blood clotting. In addition, 3 ml of venous blood was collected from the mother after delivery with sterile and disposable syringe. Hematological parameters mainly RBC parameters such as RBC count, Hgb, Hct, MCV, MCH, and MCHC were analyzed by using Sysmex KX-21 N (Sysmex Corporation Kobe, Japan) automated hematological whole blood analyzer based on direct current principle (Annex IV). Three experienced laboratory technologist performs the CBC by strictly adhering standard operating procedures.

### Data quality control

A pre-test was done to assess the integrity of questioner among 5% of the sample size at Debre Tabor General Hospital. To assure the quality of the laboratory data standard operating procedures were followed during specimen collection and CBC analysis. Thus, to avoid hemolysis after collection blood was dispensed to the wall of the test tube and properly mixed by inverting the tube gently 8–10 times. In order to avoid mix up after collection labeling was done on the sample and the request paper with the same identification number. Reagent expired date was checked before analysis of patient samples. Daily background run was done to minimize background error. The quality of socio-demographic and clinical data was maintained by daily cross-checking of collected data with maternal records and onsite supervision of the data collector during the data collection period. Training was also given for the data collectors.

### Statistical analysis

The data were cleaned, edited, checked for completeness manually and entered into Epi-info version 7.2.1.0. Then it was transferred into SPSS version 20 for analysis. Before any analysis, normal distribution of data was checked using the Kolmogorov-Smirnov and Shapiro-Wilk test. Since all parameters were not normally distributed, non-parametric tests were used. Descriptive results were summarized by percentage, frequency, median and interquartile range (IQR). The result also presented with tables. Bivariable and multivariable logistic regression was performed to assess the associated factors. Multivariate logistic regression was analyzed with backward stepwise likelihood ratio when *p*-value is less than 0.25 in bivariable binary logistic regression. The association of the independent variable with the categorical outcome variable was measured by calculating the odds ratio with a 95% confidence interval. The model fitness of the final logistic regression model was tested by using Hosmer and Lemeshow test at a *p*-value greater than 0.05. The model was adequate and fitted the data on the regression model. *P*-value of < 0.05 was considered as statistically significant.

## Results

### Sociodemographic and obstetric data of study participants

One hundred ninety-two newborn babies, which comprise (96 male and 96 females), and their mothers were enrolled in this study. During sample collection 20 newborn babies were excluded. From them, 3 newborn babies were identical twins, 10 newborn babies were from mothers with hypertension and 7 were from mothers with preeclampsia. But the excluded study participants were substituted with the next consecutive study participants. Almost 69% of newborn babies were delivered from maternal age below 30 years. Of the total newborn babies, 54.7% were born from multigravida mother. Most babies were delivered through spontaneous normal vaginal delivery 80.2% and with normal birth weight 90.6%. The median (IQR) birth weight of the study participants was 3.2(2.8–3.5) kg. The majority of newborn babies 84.9% and 67.7% were born from mothers living in urban and house-wife by their occupation, respectively. Clinical data showed that about 6.8% mothers had no antenatal care checkup. The majority of mothers 80.2% and 65.6% had taken iron and folic acid supplementation during pregnancy, respectively. About 8.9% of mothers had bleeding experience during their pregnancy (Table 1).

**Table 1:** Sociodemographic characteristics and clinical data of study participant

Maternal and newborn characteristics		Frequency	Percentage (%)
Newborn gender	Male	96	50
	Female	96	50
Newborn birth weight	Under < 2.5 kg	18	9.4
	Normal ≥2.5 kg	174	90.6
Maternal age group	≤24 years	61	31.8
	25–29 years	72	37.5
	≥ 30 years	59	30.7
Residence	Rural	29	15.1
	Urban	163	84.9
Occupation	House wife	130	67.7
	Employed	62	32.3
Educational status	Unable to read and write	33	17.2
	Primary education	40	20.8
	Secondary education	56	29.2
	Above secondary education	63	32.8
Gravida	Primi-gravida	87	45.3
	Multi-gravida	105	54.7
ANC follow up	Yes	179	93.2
	No	13	6.8
Vegetable consumption	No	48	25
	Yes	144	75
Fruit consumption	No	52	27.1
	Yes	140	72.9
Red meat consumption	No	68	35.4
	Yes	124	64.6
Mode of delivery	Normal vaginal	154	80.2
	Cesarean section	38	19.8
Bleeding during pregnancy	Yes	17	8.9
	No	175	91.1

### Prevalence of anemia among newborn babies

The cord Hgb value ranges from 4.2 – 20 g/dl with a median (IQR) value of 15 g/dL (13.9–16.2 g/dL). The overall prevalence of anemia among newborn babies was 25% (48/192) with a prevalence of 29.2 and 20.8% among male and female newborn babies, respectively. Based on severity of anemia, 89.6, 6.3, and 4.2% were mild, moderate and severe anemia, respectively. Of the total anemic newborn babies, 14.5% (7/48) had microcytic hypochromic type of anemia and 85.5% (41/48) had normocytic normochromic anemia type. High prevalence of anemia was observed in those babies born from who do not consume vegetable (41.7%) and fruits (38.5%). Based on maternal occupation, 26.9% anemic newborn babies were born from mothers who were a housewife in their occupation (Table 2). The prevalence of anemia among under and normal birth weight newborn babies was 16.7 and 25.9%, respectively.

**Table 2:** Bivariable and multivariable binary logistic regression analysis of factors associated with anemia among newborn babies

Characteristics of study participants		Anemic	Nonanemic	COR (95%CI)	AOR (95%CI)
Newborn gender	Male	28 (29.2%)		1.57(0.81–3.03)	1.47(0.74–2.90)
	Female	20(20.8%)	76 (79.2%)	1	1
ANC follow up	Yes	44 (24.6%)	135(75.4%)	1	1
	No	4 (30.8%)	9 (69.2%)	1.36(0.4–4.65)	1.69(0.34–7.42)
Iron & folic acid supplementation	No	9 (27.3%)	24 (72.7%)	1	1
	Yes	39 (24.5%)	120 (75.5%)	0.87(0.37–2.02)	1.47(0.49–4.37)
Maternal age	≤24 year	12 (19.7%)	49 (80.3%)	1	1
	25–29 year	20 (27.8%)	52 (72.2%)	1.57 (0.69–3.55)	1.7(0.67–4.35)
	≥30 year	16 (27.1%)	43 (72.9%)	1.52 (0.65–3.57)	1.22(0.42–3.58)
Educational status	Unable to read & write	9 (27.3%)	24 (72.7%)	1.02(0.39–2.62)	0.35(0.09–1.26)
	Primary school	9 (22.5%)	31 (77.5%)	0.79(0.31–1.99)	0.45(0.14–1.48)
	Secondary school	13 (23.2%)	43 (76.8%)	0.82(0.36–1.88)	0.54(0.19–1.46)
	Above secondary	17 (27.0%)	46 (73.0%)	1	1
Maternal occupation	House wife	35 (26.9%)	95 (73.1%)	1.4(0.67–2.86)	2.17(0.85–5.55
	Employed	13 (21%)	49 (79%)	1	1
Gravida	Primigravida	18 (20.7%)	69 (79.3%)	1	1
	multigravida	30 (28.6%)	75 (71.4%)	1.53(0.79–2.99)	1.45(0.72–2.89)
Vegetable consumption per week	No	20 (41.7%)	28 (58.3%)	1	1
	Yes	28 (19.4%)	116 (80.6%)	**0.34(0.17–0.69)**	**0.34 (0.17–0.69)**
Fruit consumption per week	No	20 (38.5%)	32 (61.5%)	1	1
	Yes	28 (20%)	112 (80%)	**0.4 (0.2–0.80)**	0.62 (0.25–1.49)
Red meat consumption	No	17 (25.0%)	51(75.0%)	1.0(0.51–1.98)	0.92(0.44–1.92)
	Yes	31 (25%)	93 (75%)	1	1
Maternal anemia	Yes	8 (24.2%)	25 (75.8%)	0.95(0.39–2.28)	0.79(0.29–2.13)
	No	44 (27.7%)	115 (72.3%)	1	1
Residence	Rural	9 (31%)	20 (69%)	1.43(0.60–3.39)	1.19(0.39–3.59)
	Urban	39 (23.9%)	124 (76.1%)	1	1

N.B: Bold numeric indicate significant association

### Associated factors of newborn anemia

To determine the association between the independent and outcome variable, bivariable and multivariable binary logistic regression was done. All variables were tested by bivariable binary logistic regression analysis. Based on the analysis maternal consumption habit of vegetable and fruit was significantly associated with anemia among newborn babies in bivariable logistic regression. Only four variables had a *p*-value of less than 0.25 in the bivariable analysis were fitted in multivariate logistic regression analysis to prevent the effect of a confounder. However, mothers’ vegetable consume habit (AOR =0.34, 95%CI: 0.17, 0.69) only remained a significant association of newborn anemia in multivariate binary logistic regression analysis (Table 2).

## Discussion

The present study showed that 2 (4.2%) of study participants were severely affected by anemia. This severe newbon anemia was occurred due to mothers who were not on iron and/or folic supplementation and who had bleeding history during their pregnancy. The current study also tried to figure out the prevalence of anemia among newborn babies. The overall anemia prevalence among newborn babies was 25% (95% CI: 18.9, 31.1%). According to WHO recommendation our finding showed a moderate public health problem in the study area [15]. Due to reduced oxygen-carrying capacity; anemia has a still serious public health implication that leads to newborn morbidity and mortality [16]. A number of similar findings had been reported from different countries such as in Nigeria (28.9%) [17], in New York (21%) [18], in Brazil (32.6%) [19] and in Netherland (21%) [14] that supports our findings.

However, the current study anemia prevalence among newborn babies was higher compared to study from Addis Ababa, Ethiopia showing 9% of prevalence [20]. The possible reason for this discrepancy might be due to sample size variations. In this study, 192 study participants were included, whereas a study from Addis Ababa was conducted among 89 study participants. In addition to the above, the current anemia prevalence was higher than reported from India (19.44%) [21], from United State of America (14%) [22], from Nepal (5.7%) [23] and from Iran (5.8, and 11.7%) [24, 25]. The possible explanation for this discrepancy in the prevalence of anemia across studies might be due to poor socio-economic status, and clinical characteristics of our study participants as compared to the above-listed countries.

On the other hand, the present study anemia prevalence was lower compared to the study reported from Ghana (57.3%) [26]. A lower anemia prevalence of the current study might be due to study babies born from HIV infected mothers was excluded from being sampled but Ghana’s study includes study participants born from HIV infected mothers. HIV might increase the prevalence of anemia among newborn babies in Ghana study participants as compared to the current study.

In addition to the above, our study prevalence of anemia was much lower than a study reported in Benin (61.1%) [27]. The possible difference might be attributed to that in Benin study was conducted among newborn delivered from malaria-infected mothers were included in the study, However, newborn babies born from malaria-free mothers were included in the current study. Malaria parasites transmitted to the fetus through congenital and destroyed the fetal RBC intracellular. This leads to lower Hgb value (anemia) and subsequently increases prevalence of newborn anemia in Benin study as compared to our study [28].

The other possible additional reason for the high prevalence of anemia in Benin might be due to inclusion of premature newborn babies (this study only includes full-term babies). Preterm newborns are not complete their full gestational age. The majority of iron is stored during the last trimester of pregnancy during delivery. Due to this premature babies had negative iron balance which leads development of anemia [29]. Furthermore, in the preterm newborn, kidney is immature that cannot produce sufficient amount of erythropoietin level, which results in development of anemia as compared to full-term babies [30].

Moreover, our finding anemia prevalence was lower compared to the study reported from Iran (53%) [31]. The possible reason for this discrepancy might be attributed to variation in study participants based on the mode of delivery between these studies. In Iran, all study participants were born through cesarean section, whereas the majority of our study participant was born through normal vaginal delivery. During cesarean section, there may be accidental incision of the placenta, results bleeding leading to anemia as compared to normal vaginal delivery.

Based on maternal dietary habit mothers vegetable consumption habit (AOR =0.34, 95% CI: 0.17, 0.69) during pregnancy was significantly associated with the newborn anemia in both bivariable and multivariable logistic regression which is supported by a study reported in Qatar [9]. Newborn babies born from mothers who consumed vegetables were 66% is less likely to develop newborn anemia as compared to born from mother who never consumed vegetables. This might be due to that vegetable is an important source of micronutrients such as non-heme iron and foliate that used for the newly RBC synthesis for both in the mothers as well as in the newborn babies. The fetus is an active parasite and uptake iron from maternal circulation actively and transported into fetal circulation through the placenta ready for their own Hgb synthesis which results reduce anemia development [32]. The major limitation of this study was iron test was not incorporated iron deficiency anemia may be the cause of newborn anemia. In addition, it also cannot infer direct biological causality; do not asses’ presence feto-maternal and fetoplacental hemorrhage before delivery.

## Conclusion

In general, anemia among newborn babies was a moderate public health problem in the study area. Hence, effort should be given to reduce its burden. Improving maternal nutritional status especially vegetable consumption during pregnancy had a positive impact on the prevention of newborn anemia. Based on the finding screening of anemia among newborn babies, reduce further complications.

## Data Availability

The datasets used and/or analyzed during the current study available from the corresponding author on reasonable request.

## Abbreviations

ANC: Antenatal Care
AOR: Adjusted Odds Ratio
CBC: Complete Blood Count
COR: Crude Odds Ratio
EDTA: Ethylene Diamine Tetraacetic Acid
Hct: Hematocrit
Hgb: Hemoglobin
HIV: Human Immunodeficiency Virus
IQR: Interquartile Range
MCH: Mean Cell Hemoglobin
MCHC: Mean Cell Hemoglobin Concentration
MCV: Mean Cell Volume
RBC: Red Blood Cell
UCB: Umbilical Cord Blood
UGCSH: University Gondar Compressive Specialized Hospital
WHO: World Health Organization
